# Editorial for the Special Issue on MEMS Accelerometers

**DOI:** 10.3390/mi10050290

**Published:** 2019-04-29

**Authors:** Mahmoud Rasras, Ibrahim (Abe) M. Elfadel, Ha Duong Ngo

**Affiliations:** 1 Electrical and Computer Engineering, Engineering Division, New York University Abu Dhabi, Abu Dhabi, UAE; 2 Department of Electrical and Computer Engineering, Khalifa University, Abu Dhabi, UAE; 3 Hochschule für Technik und Wirtschaft Berlin, University of Applied Sciences, Treskowallee 8, 10318 Berlin, Germany; 4 Fraunhofer Institute for Reliability and Microintegration IZM, Department Wafer Level Integration, Group Leader Microsensors Technology, Gustav-Meyer-Allee 25, 13355 Berlin, Germany

Micro-Electro-Mechanical Systems (MEMS) devices are widely used for motion, pressure, light, and ultrasound sensing applications. They are also used as micro switches and micro actuators in control applications. Research on integrated MEMS technology has undergone extensive development driven by the requirements of compact footprint, low cost, and increased functionality. Accelerometers are among the most widely used sensors implemented in MEMS technology. MEMS Accelerometers are showing a growing presence in almost all industries, ranging from consumer electronics to transportation and from games and entertainment to healthcare. Their MEMS embodiment has evolved from single, stand-alone devices to the integrated, 6-axis and 9-axis inertial motion units that are available on the market today. A traditional MEMS accelerometer employs a proof mass suspended to springs, which displaces in response to an external acceleration. A single proof mass can be used for one- or multi-axis sensing. A variety of transduction mechanisms have been used to detect the displacement. They include—capacitive, piezoelectric, piezoresistive, thermal, tunneling, and optical. Capacitive accelerometers are widely used due to their DC measurement interface, thermal stability, reliability, and low-cost. However, they are sensitive to electromagnetic field interferences and have poor performance for high-end applications (e.g., precise attitude control for satellites). Over the past three decades, steady progress has been made in the area of optical accelerometers for high-performance and high-sensitivity applications but several challenges are still to be tackled by researchers and engineers to fully realize Opto-Mechanical Accelerometers, such as chip-scale integration, scaling, low bandwidth, etc. Currently, optical technologies are still used in navigation systems and tactical guidance. New applications have been enabled by low-cost MEMS sensors, and significant progress has been made in the past few years in terms of their reliability. MEMS accelerometers are now accepted in high-reliability environments, and are even starting to replace optical and other established technologies.

This Special Issue on “MEMS Accelerometers” includes research papers, short communications, and review articles. There are 16 papers published covering the design, fabrication, modeling and applications of MEMS accelerometers. Half of the papers discuss accelerometer integration [1,2], piezoresistive sensing [3,4] multi-axis accelerometers, and review current technologies [4,5,6]. Three papers investigate MEMS accelerometer multi-physics modeling [7,8,9,10]. The rest of the papers are focused on the application domains, including environmental monitoring [11] and WiFi positioning [12]. Healthcare monitoring, positioning and daily activity monitoring are discussed in [13,14], while wearable body sensors for patients with gait impairments and the classification of horse gaits for self-coaching are covered in [15,16].

On the device design and integration, H. Liu et al. [1] demonstrate a hybrid-integrated, high-precision, vacuum accelerometer based on field emission. It shows a sensitivity of 3.081 V/g, the non-linearity is 0.84% in the acceleration range of −1 g to 1 g, while the average noise spectrum density value is 36.7 μV/Hz in the frequency range of 0–200 Hz. H. Liu et al. [2] develop a differential capacitive accelerometer based on low-temperature co-fired ceramic (LTCC) technology for harsh-environment applications. The device has a full-scale range of 10 g with a sensitivity of 30.27 mV/g. X. Hu et al. [3] report on a family of silicon-on-insulator (SOI)–based high-g MEMS piezoresistive sensors for the measurement of accelerations up to 60,000 g. In this device, four piezoresistors are connected in a Wheatstone bridge to measure acceleration. X. Zhao et al. [4] also develop a silicon-on-insulator (SOI) piezoresistive, three-axis acceleration sensor with demonstrated sensitivities along x-axis, y-axis, and z-axis of 0.255 mV/g, 0.131 mV/g, and 0.404 mV/g, respectively. A thermal convection-based accelerometer is fabricated and characterized by J. Kim et al. [5]. They investigate the impact of cavity volume, gas medium density and viscosity with a focus on the Z-axis response. Z. Mohammed et al. [6] provide an in-depth review of monolithic multi-axis capacitive MEMS accelerometers, including a detailed analysis of recent advancements aimed at addressing various challenges such as size, noise floor, cross-axis sensitivity, and process aware modeling.

As for multi-physics modeling, X. Dong et al. [7] develop an experimental method for measuring the parasitic capacitance mismatch in a MEMS accelerometer. This result is helpful for improving bias performance and the scale factor. F. Wang et al. [8] report on the design, modeling, and fabrication of an elastic-beam delay element. Chen D. et al. [9] propose using a fifth-order ΣΔ closed-loop interface for a capacitive MEMS accelerometer that includes a digital built-in self-testing feature. By a single-bit ΣΔ-modulation, the noise and linearity of excitation is effectively improved, and a higher detection level for distortion is achieved. Yang Z. et al. [10] show that the angular-rate sensing based on mode splitting offers good suppression of Kerr noise. They demonstrate that at an angular rate of 5 × 106°/s, a Kerr noise of 1.913 × 10^−5^ Hz is measured which corresponds to an angular rate deviation of 9.26 × 10^−9^°/s.

As for MEMS accelerometer applications, Tian B. et al. [11] design a probe for marine environmental monitoring to estimate the ocean turbulent kinetic energy dissipation rate. They achieve a sensitivity of 3.91 × 10^−4^ (Vms^2^)/kg over a measurement range of 10^−8^–10^−4^ W/kg. Lai M. et al. [12] study a large amount of raw data measured by a MEMS accelerometer-based wrist-worn device. This device is used to monitor different levels of physical activities (PAs) for subjects wearing it continuously 24 h a day. Lin W. et al. [13] develop a method using multi-mounted devices to construct a lightweight site-survey radio map (LSS-RM) for WiFi positioning. Their experimental results show that their method can reduce the time required to construct a WiFi-received signal strength index (RSSI) radio map from 54 min to 7.6 min. Yuan C. et al. [14] propose a novel framework for fault-tolerant visual-inertial odometry (VIO) navigation and positioning. Qiu. S. et al. [15] show promising results for a low-cost, intelligent and lightweight wearable gait analysis platform based on body IMU sensor networks. They have assembled the IMU from accelerometers/gyroscopes chipsets. A multi-sensor fusion algorithm is used to estimate the gait parameters. The method has great potential as an auxiliary for medical rehabilitation assessment. Lee J. et al. [16] investigate the classification of horse gaits using MEMS inertial sensor technology with the goal of developing a horse-gait self-coaching platform based on machine learning methods. In the experimental setup, the authors employ a camera-less 3D human motion measurement system based on state-of-the-art MEMS inertial sensors, biomechanical models, and sensor fusion algorithms.

We would like to thank all the authors for submitting their original papers to this special issue. Special thanks are also due to all the reviewers for their dedicated efforts in helping to improve the quality of the submitted papers. Finally, we are grateful to Ms. Mandy Zhang and the MDPI team for all their editorial assistance. 
